# QmRLFS-finder: a model, web server and stand-alone tool for prediction and analysis of R-loop forming sequences

**DOI:** 10.1093/nar/gkv344

**Published:** 2015-04-16

**Authors:** Piroon Jenjaroenpun, Thidathip Wongsurawat, Surya Pavan Yenamandra, Vladimir A. Kuznetsov

**Affiliations:** Department of Genome and Gene Expression Data Analysis, Bioinformatics Institute, Agency for Science, Technology and Research (A*STAR), 30 Biopolis street, #07-01, Singapore 138671

## Abstract

The possible formation of three-stranded RNA and DNA hybrid structures (R-loops) in thousands of functionally important guanine-rich genic and inter-genic regions could suggest their involvement in transcriptional regulation and even development of diseases. Here, we introduce the first freely available R-loop prediction program called Quantitative Model of R-loop Forming Sequence (RLFS) finder (QmRLFS-finder), which predicts RLFSs in nucleic acid sequences based on experimentally supported structural models of RLFSs. QmRLFS-finder operates via a web server or a stand-alone command line tool. This tool identifies and visualizes RLFS coordinates from any natural or artificial DNA or RNA input sequences and creates standards-compliant output files for further annotation and analysis. QmRLFS-finder demonstrates highly accurate predictions of the detected RLFSs, proposing new perspective to further discoveries in R-loop biology, biotechnology and molecular therapy. QmRLFS-finder is freely available at http://rloop.bii.a-star.edu.sg/?pg=qmrlfs-finder.

## INTRODUCTION

The R-loop is a three-stranded nucleic acid structure that is co-transcriptionally formed RNA–DNA hybrid between a nascent guanine-rich RNA transcript segment and a DNA template whilst leaving the non-template DNA strand in a single-stranded conformation. R-loops lie at the interface of multiple biological processes, including RNA transcription and processing, chromatin interactions, DNA damage, mutagenesis as well as cell proliferation and differentiation. Altered R-loops balance can impair R-loop-mediated processes, resulting in mutagenesis and genome instability and possibly leading to various diseases. The targeting of RNA–DNA hybrids in R-loops using small molecules has the potential to be clinically important. Thus, these types of strategy are currently under development (1).

The systematic detection and prediction of R-loops are key issues for structural and functional characterization of R-loops (2). Recently, the DNA–RNA immunoprecipitation sequencing (DRIP-seq) method has been developed for detecting RNA–DNA hybrids at the genome-wide scale (3). This method has been shown to detect more than 4000 possible R-loops of the human genome, specifically in stem-cell like cells Ntera2 (3). However, these findings still have limitations which can be associated with the use of only a single cell type, the environmental context (retinoid acid–induced initiation of the cells for differentiation), the sensitivity and specificity of S9.6 antibody, the performance of selected cocktails of restriction enzymes, technical errors and biological variations. For these reasons, DRIP-seq is not yet sufficient without independent testing to identify/map all actual functional R-loops and to refine their boundaries in the genome. The computational models of Quantitative Model of R-loop Forming Sequences (RLFSs) are based on the assumptions related to composition and structure of nucleic acid sequence data. Theoretically, they could predict all possible R-loops in the genome.

In 2011, we published a quantitative structural model of RLFSs (4), whose parameters were optimized based on publicly available *in vitro* and *in vivo* data. Here, we extended our previously published computational structural model for RLFS prediction (4) and used this generalized model, which we termed as QmRLFS-finder, to develop a pipeline for predicting the structure and sequence location of RLFSs. QmRLFS-finder is an R-loop prediction tool that can be applied to any DNA or RNA sequence. This generalized analytical tool allows the user to search for RLFSs in the sequences without specification of the sequence origin, cell type or organism context. We demonstrate the accuracy and predictive power of QmRLFS-finder. For the convenience of further analysis, the program generates results in several formats that can be used for immediate viewing using the UCSC Genome Browser. Finally, we provide examples of the usage of our program and interpretations of the results.

## MATERIALS AND METHODS

### Generalized structural motif models of RLFSs

Here, we describe the extension of our original quantitative structural model for RLFS prediction, reported in (4), and then describe how we used that generalized model to develop a pipeline for predicting the structure and location of RLFSs, which was finally implemented in the QmRLFS-finder program.

Briefly, our computational models of RLFS (4) has identified three structural features in DNA sequences, including a short G-cluster-rich region responsible for initiating R-loop formation (R-loop initiation zone or RIZ), a structurally non-specified linker (linker) and a downstream region that is relatively long and has a high G-density R-loop elongation zone (or REZ). The three zones (or sequence elements) constitute the RIZ-Linker-REZ configuration and form the basis of our computational RLFS prediction model. Such sequence elements and their configuration in the non-template DNA sequence have been proposed in (5) based on biochemical and molecular biology studies of the roles of the G-clusters and high G-density sequences in transcriptional R-loop formation. Using the characteristics of empirical R-loop sequence models (5), the computational model (4) predicts the locations of RLFS in the genes of human genome.

Here, we generalized our original RLFS model (4). In additional to the previous quantitative structural model of RLFS, the generalized model can also use two linked G-clusters and increase the number of contiguous-Gs to four guanines as a RIZ, as it was reported based on *in vitro* findings (5). Thus, we can improve the sensitivity of the prediction model via incorporating an additional sequence composition for RIZ into the RLFS model (4).

The main aim of the QmRLFS-finder program is to predict the presence and location of the RLFSs in nucleotide sequences. The RLFSs are identified using a simple pattern-based rule by matching sequences of the form

(m1) \begin{equation*} \underbrace {G_{n1} N_x G_{n1} N_x G_{n1} }_{{\rm RIZ}},\underbrace {N_y }_{{\rm linker}},\underbrace {N_z }_{{\rm REZ}}\left\{ {\begin{array}{*{20}c} {{\rm RIZ}{\rm } \ge 50\% {\rm G}} \\ {{\rm REZ} \ge 40\% {\rm G}} \\ \end{array},} \right. \end{equation*}

(m2) \begin{equation*} \underbrace {G_{n2} N_x G_{n2} }_{{\rm RIZ}},\underbrace {N_y }_{{\rm linker}},\underbrace {N_z }_{{\rm REZ}}\left\{ {\begin{array}{*{20}c} {{\rm RIZ} \ge 50\% {\rm G}} \\ {{\rm REZ} \ge 40\% {\rm G}} \\ \end{array},} \right. \end{equation*}

where *G_n_*_1_ and *G_n_*_2_ are the guanine cores that can occur with different numbers of G-residues (*n*1 ≥ 3 and *n*2 ≥ 4) in RIZ sequence feature. The RIZ in (m1) contains three G-clusters, and the RIZ in (m2) contains two G-clusters. The symbol *N_x_* denotes a sequence with non-specified composition of nucleotides, where the number of nucleotides (*x*) 1 ≤ *x* ≤ 10. Additionally, the RIZ must contain at least 50% guanine content in both models. For the Linker sequence feature, the symbol *N_y_* denotes an arbitrary nucleotide composition sequence number (*y*) 0 ≤ *y* ≤ 50 which does not affect R-loop extension (6). For the REZ sequence feature, the symbol *N_z_* denotes an arbitrary nucleotide composition sequence number (*z*) 100 ≤ *z* ≤ 2000. REZ requires at least 40% guanine content which is considered as an important feature to maintain R-loops formation (6). By our model, the REZ region can also include the G-cluster regions.

Our generalized quantitative structure RLFS predictive model (m1) and (m2) is not *a priori* limited by any other sequence composition constraints or pre-selected regulatory signals, including CpG islands, repeats, gene loci or genome architectures.

### QmRLFS-finder algorithm

**Figure 1.**
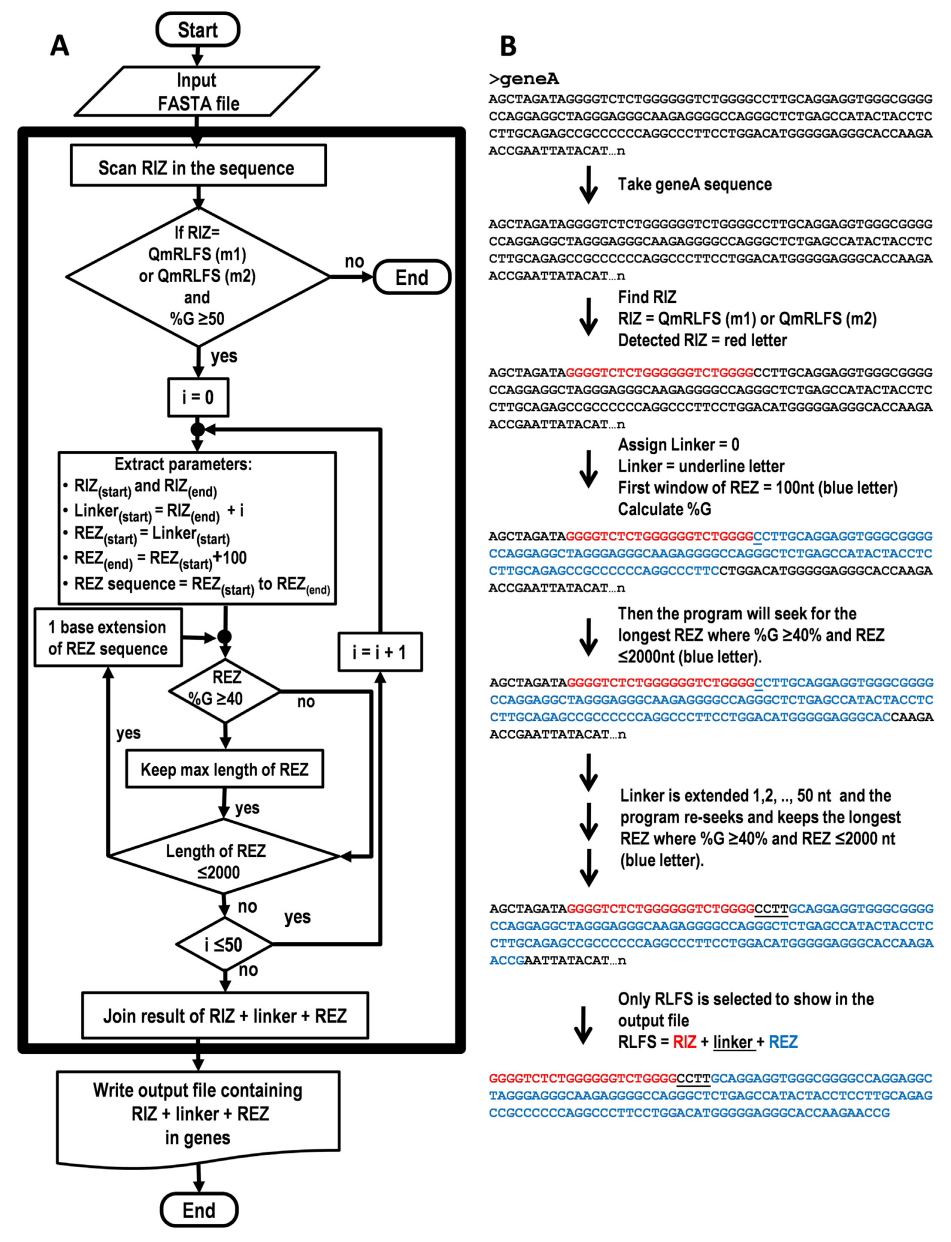
Algorithm outlining RLFS prediction. (A) a flowchart of QmRLFS-finder algorithm. (B) An example of RLFS prediction step with a DNA query sequence.

The QmRLFS-finder algorithm consists of the following steps (a flowchart of QmRLFS-finder algorithm and an example is shown in Figure 1A-B):

- Input: QmRLFS-finder takes DNA (or RNA) sequences in FASTA format and searches for a possible RIZ according to the input QmRLFS model (m1) or (m2), linker and REZ.
- Searching for a possible RIZ: The first step of the program searches for sequences that satisfy sequences of the following form: QmRLFS model (m1) or (m2). Then, the % G content is calculated for the sequence. The sequence must contain at least 50% G. The location of the RIZ (start and end positions) and the number of G-clusters are mapped and counted accordingly.
- Searching for the linker and REZ: The end position of the RIZ is used as the start position of the linker segment called linker_[start]_. This linker sequence region can vary from 0 to 50 nucleotides. In the first iteration, linker_[start]_ is 0, and the REZ window is initially set at 100 nucleotides. The % G content of the first REZ window is calculated; sequences with at least 40% G (threshold) are considered and stored for the next search. The window is expanded from 100 to 101, 102, …, 2000 nucleotides to find the longest REZ with a % G content greater than or equal to the model threshold level. The next iteration (linker_[start]_ +1) is started and performed following the same procedure. After all iterations have finished, only the longest REZ with a % G content greater than or equal to the threshold level of the model will be kept for the last step.
- Output: QmRLFS-finder produces reports in four output formats: an RLFS table, FASTA, BED and CUSTOM TRACK.

**Figure 2.**
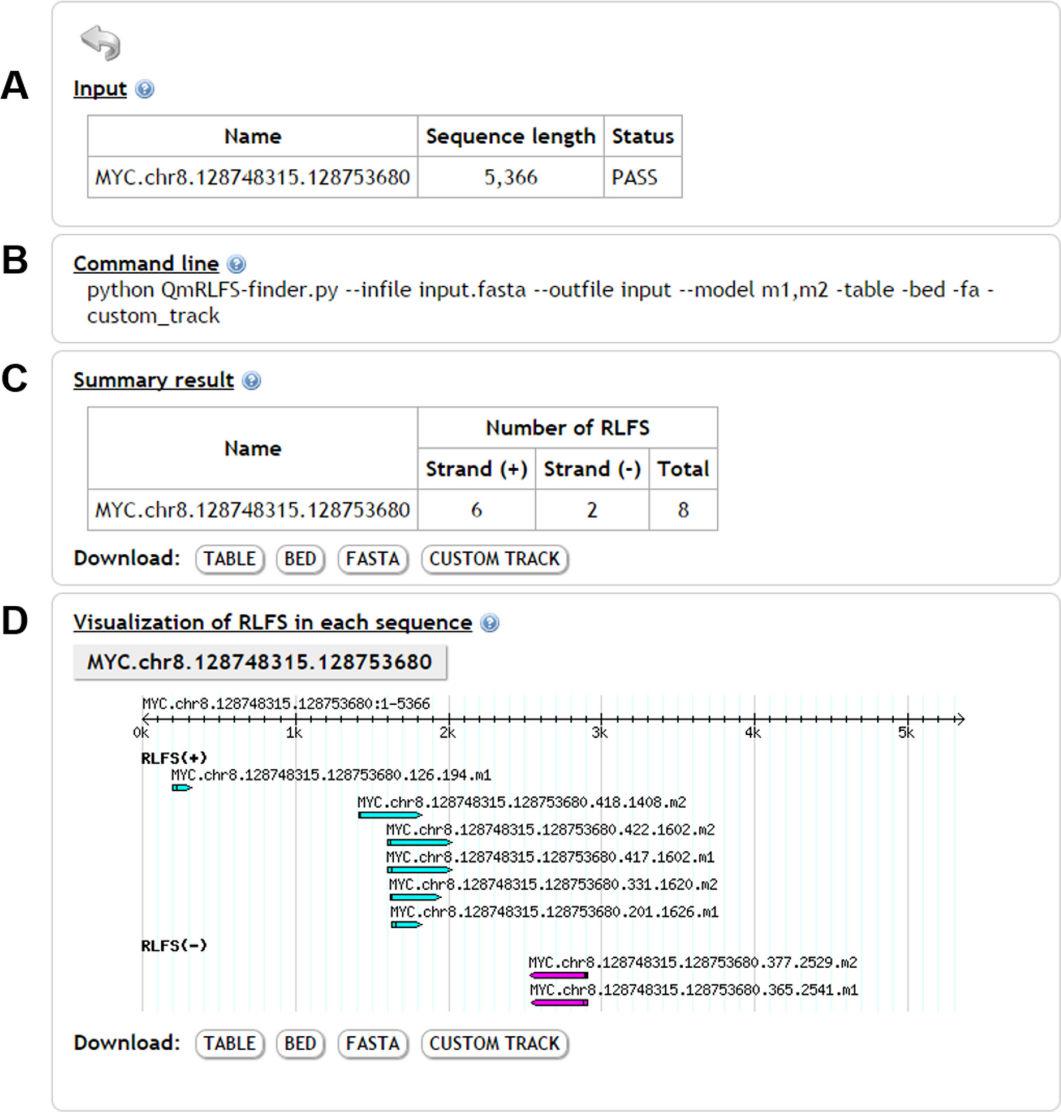
An example of RLFS prediction using the QmRLFS-finder web page. In this example, the human *MYC* gene is used as the input. (A) General information about the input. The status is ‘PASS’, indicating that the input is a DNA or RNA sequence and is in FASTA format. QmRLFS-finder allows only nucleotide sequences containing ‘A’, ‘T’, ‘C’, ‘G’, ‘U’ and ‘N’. (B) A command in the QmRLFS-finder program for the particular query. (C) A summary result for the number of RLFSs in the *MYC* gene according to each strand. Four output files are provided for download. (D) A graphical representation of a map of the RLFS locations in the input sequence.

**Figure 3.**
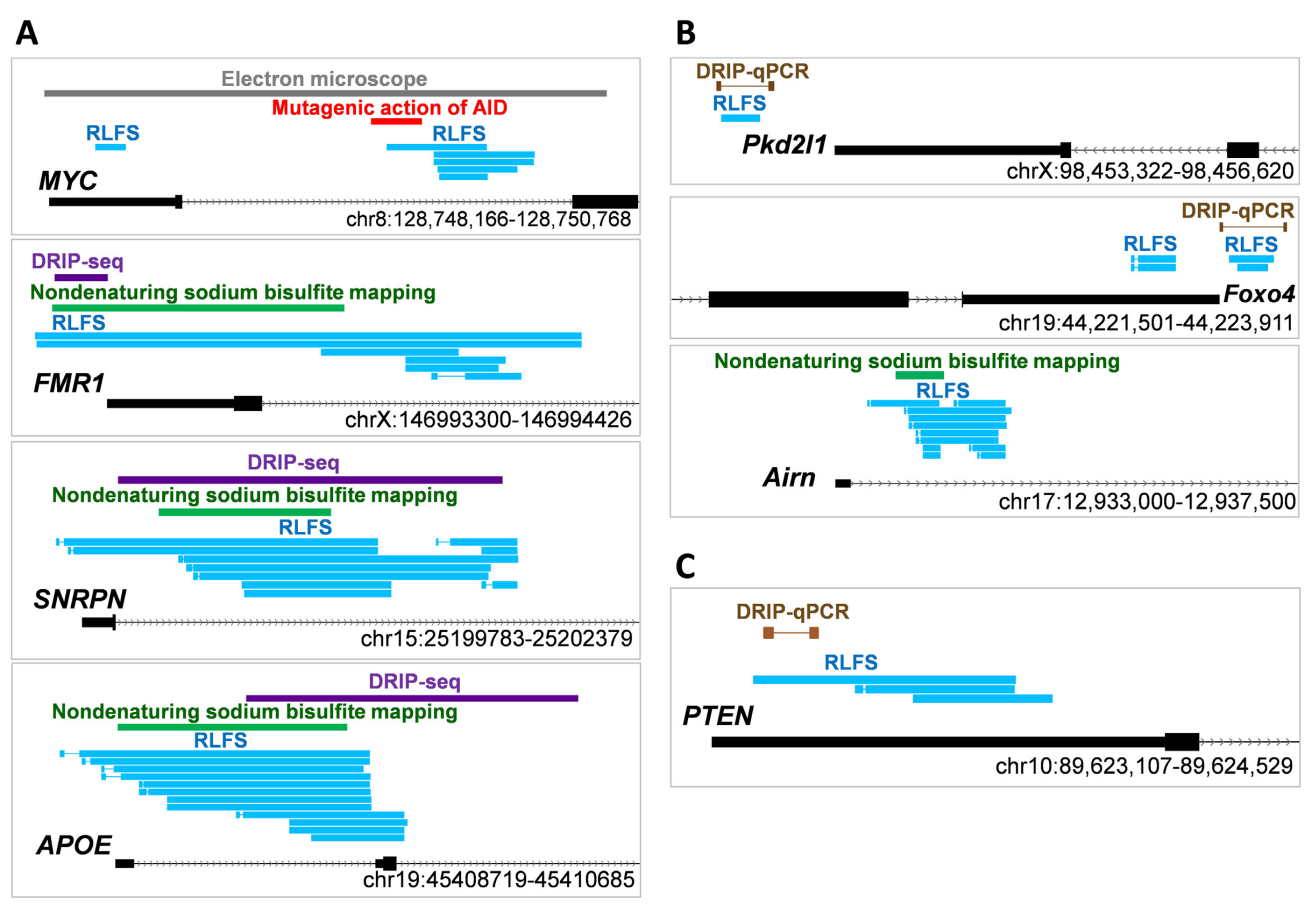
The consistency in RLFS prediction by QmRLFS-finder using models (m1) and (m2) and R-loop defined by experimental methods from independent studies. (**A**) Mapping of predicted RLFSs and R-loops defined by at least two experimental methods in four human genes, *MYC, FMR1, SNRPN* and *APOE*. (**B**) Mapping of predicted RLFSs and the R-loops defined by experimental method in three mouse genes, *Pkd2l1, Foxo4* and *Airn*.

#### Design and implementation

QmRLFS-finder is developed as both a web server and stand-alone tool for the fast prediction of RLFSs. QmRLFS-finder is implemented in Python and can be run under OS X, Windows, Linux and Unix. The web server runs on Linux system (Intel(R) Xeon(R) CPU E5-2670 v2 @ 2.50GHz).

**Table 1:** Comparison of QmRLFS-finder prediction with experimental R-loop detection

Gene	Cell line	Reference	R-loop identification method	R-loop forming signal	RLFS predicted by QmRLFS-finder
Immunoglobulin*	human epithelial carcinoma	(12)	R-loop foot-printing	+	+
*MYC*	*In vitro*	(7,8)	electron microscopy and mutagenic action of AID	+	+
*BCL6*	*In vitro*	(13)	electron microscopy	+	+
*RHOH*	*In vitro*	(13)	electron microscopy	+	+
ACTB**	Hela	(14)	DRIP-qPCR	+	+
*FMR1*	Fibroblast/Fragile X Syndrome patient GM03200	(9,10)	DRIP-qPCR and R-loop foot-printing	+	+
*SNRPN*	Ntera2	(11)	DRIP-seq and R-loop foot printing combined to RNase H digestion	+	+
*HK2*	Ntera2	(11)	DRIP-seq	+	+
*CHTF8*	Ntera2	(11)	DRIP-seq	+	+
*CIRH1A*	Ntera2	(11)	DRIP-seq	+	+
*APOE*	Ntera2	(11)	DRIP-seq and R-loop foot-printing combined to RNase H digestion	+	+
FHIT***	B-cell	(15)	Overexpressed or knocked down RNase H	+	+
*FLC*	Arabidopsis	(18)	DRIP-qPCR and R-loop foot-printing	+	−
Pkd2l1**	Mouse testes	(17)	DRIP-PCR	+	+
Foxo4**	Mouse testes	(17)	DRIP-PCR	+	+
*Airn*	Mouse embryonic stem cell	(11)	R-loop foot-printing	+	+
*C9orf72*	*In vitro*	(16)	RNase H digestion	+	+
*JTB*	SKOV3	in-house study	DRIP-qPCR	−	−
*PPMD1*	SKOV3	in-house study	DRIP-qPCR	−	−
*TP53*	SKOV3	in-house study	DRIP-qPCR	−	−
*PBX1*	SKOV3	in-house study	DRIP-qPCR	−	+
*PTEN*	SKOV3	in-house study	DRIP-qPCR	+	+

+: positive signal; −: negative signal; *switch region; **3′ end; ***fragile site FRA3B on chromosome 3.

### Experimentally defined R-loop data

To validate the predictive power of QmRLFS-finder, experimentally defined R-loops in 22 genes and/or in approximate vicinity of the genes were collected from previous publications (references in Table 1) and from in-house validation. It includes 12 human genes (*MYC* (7,8), *FMR1* (9,10), *HK2* (11),*SNRPN* (11), *APOE* (11), *CHTF8* (11), *CIRHA* (11), human immunoglobulin (12), *BCL6* (13), *ACTB* (14), *FHIT* (15), *C9orf72* (16)). Mapping of predicted RLFSs and R-loops defined by at least two experimental methods in four human genes (*MYC, FMR1, SNRPN* and *APOE*). Mapping of predicted RLFSs and R-loops defined by at least two experimental methods in four human genes (*MYC, FMR1, SNRPN* and *APOE*). Mapping of predicted RLFSs and the R-loops defined by experimental methods in three mouse genes (*Airn* (11), *Pkd2l1* (17) and *Foxo4* (17)) and one Arabidopsis gene (*FLC* (18)). For in-house validation, we used the DRIP-qPCR method to attempt to validate R-loop formations in *PTEN* (a tumour suppressor gene predicted by QmRLFS-finder as RFLS-positive) as well as in four RLFS-negative gene regions (Supplementary data).

### Performance evaluation

The performance of QmRLFS-finder was assessed using experimental data summarized in Table 1. Standard formulas of accuracy, sensitivity and specificity are also reported in Supplementary data.

## RESULTS

### Web Server

#### The features of QmRLFS-finder

We developed QmRLFS-finder using QmRLFS (Quantitative Models of RLFS) model (generalized RLFS prediction model; the Materials and Methods section) to (i) search for the presence of possible RLFSs, (ii) report RLFS locations in input sequences and (iii) produce standards-compliant output files for further analysis and visualization.

The QmRLFS-finder web server is accessible from http://rloop.bii.a-star.edu.sg/?pg=qmrlfs-finder with any standard Internet browser (Google Chrome, Safari 6+, Mozilla Firefox 5+, Internet Explorer 8+ and Opera 11+). We recommend the use of Google Chrome browser, because this site was mainly developed in that browser. We also provide a QmRLFS-finder program (stand-alone version) for searching RLFSs in a large input sequence such as a human chromosome or genome. The source code, command-line usage and examples of the QmRLFS-finder applications are available online at http://rloop.bii.a-star.edu.sg/?pg=qmrlfs.

### Server input

The QmRLFS-finder web server allows users to enter single or multiple DNA (or RNA) sequences in FASTA format and these can be either pasted (up to 300 000 letters) or uploaded as a file (up to 300 kB). To facilitate using the tool, help page links are available within the interface. The tool offers options to choose different QmRLFS models, according to (m1), (m2), or (m1, m2) (default), for predicting RLFSs. Additionally, to facilitate viewing RLFS results in the UCSC Genome Browser, the ‘Optional’ tool allows users to define the chromosome localizations of the input sequence and to define its strand. The defined chromosome coordinates can be used to re-calculate the RLFSs positions in a given chromosome.

### Server output

The results page of QmRLFS-finder displays the items in the following order (see Figure 2):

- ‘Input’ shows the list of input sequences, their lengths and their status (whether the input sequences are appropriate).
- ‘Command line’ shows an example command line from the QmRLFS-finder program.
- ‘Summary result’ shows the number of RLFSs in a particular input sequence and provides download links for four output file formats: an RLFS table, FASTA, BED and CUSTOM TRACK. The RLFS table file contains detailed information about the RIZ, linker and REZ features, including the names of the input sequences, their start and stop locations, the lengths of the RIZ and REZ, the number of G clusters and the sequence of each feature. The FASTA file contains the names of the input sequences and the RLFS sequences. The BED and CUSTOM TRACK files (https://genome.ucsc.edu/goldenPath/help/customTrack.html) contain the locations of the RLFS, RIZ and REZ in the input sequence.
- ‘Visualization of RLFS in each sequence’ shows a graphic of RLFS locations mapped to a given input sequence. Download links are provided.

### Example

To illustrate the usage of QmRLFS-finder, we submitted DNA sequence of human *MYC* gene downloaded from UCSC genome browser (hg19: chr8:128748315–128753680) as an input sequence. Figure 2 shows the output of RLFSs in *MYC* gene using QmRLFS-finder. As shown in Figure 2A, the input has only one sequence with 5366 bp in length. We found a total of eight RLFSs in *MYC* gene including six RLFSs on positive strand and two RLFSs on negative strand (Figure 2C). The graphical result, in Figure 2D, represents a map of the RLFS locations along the input sequence. In this gene, two out of the three loci show overlapping of multiple RLFSs. These loci can be termed as ‘RLFS cluster’ which, according to our definition, contains at least two overlapping RLFSs. A higher number of RLFSs in the RLFS clusters implied a higher propensity of R-loop initiation and formation in a given locus. We propose that detailed analysis of predicted RLFS clusters should be important and targets for further studies.

## VALIDATION

### Contribution of experimentally supported RLFS model 2 in a generalized structural QmRLFS model

We generalized our previous reported RLFS model (m1; the Materials and Methods section) by incorporating an additional criterion of R-loop initiation zone (RIZ) formation as it was observed in *in vitro* studies (5) and described in ‘m2’ model in the Materials and Methods section. The model m2 predicts RLFSs for 597 additional genes, which cannot be predicted by m1. Our generalized structural QmRLFS model (called ‘m1, m2’) can predict the same regions of RLFSs reported in our previous study (4) as well as new RLFS regions and new RLFS-positive genes. According to the extended QmRLFS model, the number of RLFS-positive RefSeq genes consists of 19 573 (75.7%) out of 25 844 RefSeq genes.

### Evaluation of QmRLFS-finder prediction power

Twenty two genes are used to validate the predictive performance of QmRLFS-finder. We collected 17 genes reported to contain experimentally defined locations of the R-loops that had been verified (Table 1). Currently, this is a set of all experimentally defined and studied RLFSs detected by different methods at a single gene level carried out in the 16 mammalian and single plant genes. Other five cases in Table 1 were experimentally validated by us using the DRIP-qPCR method (Supplementary Figure S1 and Supplementary Table S2). The theoretically expected loci of RLFS preferential location in genes were used for identification of QmRLFS-positive and QmRLFS-negative control loci within gene territories. According to QmRLFS-finder, we selected three RLFS-negative regions including the 5′ UTR downstream transcription start site of *JTB* gene, the 3′ UTR of gene *PPM1D*, and in the intronic region of *TP53* and two RLFS-positive regions, selected in the 5′ UTR of *PTEN* gene and in the intronic region of *PBX1* gene. DRIP-qPCR signals were negative in selected *JTB, PPM1D* and *TP53* loci, detected in cells of ovarian cancer cell line SKOV3, indicating the lack of RNA–DNA hybrid formation. DRIP-qPCR assay was positive in the QmRLFS-positive predicted locus in *PTEN* locus, and was negative in the *PBX1* locus.

It is very unlikely that the 20 of 21 computationally predicted RLFS loci can be co-localized with 20 independently detected RLFS loci located randomly within any of 21 selected mouse/human genes (Table 1). Indeed, a probability of the co-localization of QmRLFS-predicted locus and random sequence with typical RLFS length within a gene region was estimated for 20 of 21 random humans and mice genes and confirmed our assumption. According to binomial probability function, such co-occurrence event could be observed by chance with the probability less than 10^-11^ (‘Non-random mapping of the QmRLFS models and observed RLFS at a single gene level analysis’ in Supplementary data).

To evaluate the prediction performance, we compared the frequencies of experimentally defined R-loop with those present/absent RLFSs predicted by QmRLFS-finder (Table 1). QmRLFS-finder shows 91% in accuracy, 94% in sensitivity and 75% in specificity (Supplementary data).

The genome scale comparison was performed using DRIP-seq data (3). These data, however, have limitations on cell-line and specific condition but nevertheless, we could use the data to evaluate performance of prediction specifically for Ntera2 cell-line. We overlapped (at least one common base pair) the DRIP-seq defined regions (RNA–DNA hybrid regions) with RLFS predicted by QmRLFS-finder. Of 4181 DRIP-seq defined regions, 3311 regions (79.2% in sensitivity) overlap RLFSs. In addition, to estimate our prediction specificity and accuracy, we generated computationally ‘DRIP-seq-negative regions’ data by randomly sampling 4181 loci (100 times) that did not contain any DRIP-seq defined signal. QmRLFS-finder showed 88.68 ± 0.44% in specificity and, finally, 83.93 ± 0.22% in accuracy.

### Consistency between predicted RLFS and experimentally defined R-loops

Here we present overlapping of RLFSs predicted by QmRLFS-finder and experimentally defined R-loops of human and mouse genes. Figure 3A shows the examples of mapping of RLFS and experimentally defined R-loop regions in four studied human genes (*MYC* (7,8)*, FMR1* (9,10)*, SNRPN* (11) and *APOE* (11)). Figure 3B shows the RLFSs predicted by QmRLFS-finder and the locations of experimentally defined R-loops in three mouse genes (*Pkd2l1* (17), *Foxo4* (17) and *Airn* (11)). Beside previous reports of R-loops, we validated QmRLFS-finder predicted RLFS in the downstream promoter region of *PTEN*. Figure 3C shows the RLFS mapping, found by DRIP-qPCR; this location consists of the prediction provided by QmRLFS-finder for *PTEN* gene.

## DISCUSSION

QmRLFS-finder provides a user-friendly web server and stand-alone tool for rapid and accurate prediction of RLFSs in DNA or RNA sequences. The predictions by QmRLFS-finder showed strong agreement with existing genes and genome scale experimentally determined R-loops. However, in the future, a comprehensive evaluation of the reported experiments and generation of new experimental genome-wide R-loop measurements should be carried out and help in specifying the estimation of sensitivity, specificity and accuracy of both experimental approaches and RLFS predictions.

We foresee many useful applications of RLFS predictive tools in growing research areas in academia, medicine and industry. In addition to these applications, the comprehensive prediction of RLFSs with QmRLFS-finder might be useful for evaluating the ability of nucleotide sequences to form R-loops in both *in vivo* and *in vitro* systems. QmRLFS-finder has a possibility of implementing RLFS predictive models to study R-loop formation phenomena in diverse genomes and study a comparative evolution of R-loops, which is of great theoretical, experimental and practical interest.

## SUPPLEMENTARY DATA

Supplementary Data are available at NAR Online.

SUPPLEMENTARY DATA
