# QmRLFS-finder: a model, web server and stand-alone tool for prediction and analysis of R-loop forming sequences

**DOI:** 10.1093/nar/gkv974

**Published:** 2015-09-22

**Authors:** Piroon Jenjaroenpun, Thidathip Wongsurawat, Surya Pavan Yenamandra, Vladimir A. Kuznetsov

*Nucl. Acids Res*. 43 (W1): W527–W534. doi:10.1093/nar/gkv344

The authors wish to make the following corrections to their article:

In Table 1, the column heading ‘Cell line’ should be replaced by ‘Cell line/Cell type’. In the first line of the table, the ‘human epithelial carcinoma’ cell type is incorrect and should be ‘primary mammalian B lymphocytes’. A new Table 1 is presented below.

**Table 1.** Comparison of QmRLFS-finder prediction with experimental R-loop detection

Gene	**Cell line/Cell type**	Reference	R-loop identification method	R-loop forming signal	RLFS predicted by QmRLFS-finder
Immunoglobulin*	**Primary mammalian B lymphocytes**	(12)	R-loop foot-printing	+	+
*MYC*	*In vitro*	(7,8)	electron microscopy and mutagenic action of AID	+	+
*BCL6*	*In vitro*	(13)	electron microscopy	+	+
*RHOH*	*In vitro*	(13)	electron microscopy	+	+
ACTB**	Hela	(14)	DRIP-qPCR	+	+
*FMR1*	Fibroblast/Fragile X Syndrome patient GM03200	(9,10)	DRIP-qPCR and R-loop foot-printing	+	+
*SNRPN*	Ntera2	(11)	DRIP-seq and R-loop foot printing combined to RNase H digestion	+	+
*HK2*	Ntera2	(11)	DRIP-seq	+	+
*CHTF8*	Ntera2	(11)	DRIP-seq	+	+
*CIRH1A*	Ntera2	(11)	DRIP-seq	+	+
*APOE*	Ntera2	(11)	DRIP-seq and R-loop foot-printing combined to RNase H digestion	+	+
FHIT***	B-cell	(15)	Overexpressed or knocked down RNase H	+	+
*FLC*	Arabidopsis	(18)	DRIP-qPCR and R-loop foot-printing	+	−
Pkd2l1**	Mouse testes	(17)	DRIP-PCR	+	+
Foxo4**	Mouse testes	(17)	DRIP-PCR	+	+
*Airn*	Mouse embryonic stem cell	(11)	R-loop foot-printing	+	+
*C9orf72*	*In vitro*	(16)	RNase H digestion	+	+
*JTB*	SKOV3	in-house study	DRIP-qPCR	−	−
*PPMD1*	SKOV3	in-house study	DRIP-qPCR	−	−
*TP53*	SKOV3	in-house study	DRIP-qPCR	−	−
*PBX1*	SKOV3	in-house study	DRIP-qPCR	−	+
*PTEN*	SKOV3	in-house study	DRIP-qPCR	+	+

+: positive signal; −: negative signal; *switch region; **3′ end; ***fragile site FRA3B on chromosome 3.

The results and conclusion of the article are not affected and remain valid. The authors apologise to the readers for the inconvenience caused.

