# Supplementary material for: QmRLFS-finder: a model, web server and stand-alone tool for prediction and analysis of R-loop forming sequences
Source: Nucleic Acids Res. 2015 Apr 16;43(Web Server issue):W527–34. doi: 10.1093/nar/gkv344 (PMC4489302; doi:10.1093/nar/gkv344)
Supplement: SUPPLEMENTARY DATA [file supp_43_W1_W527__index.html]

QmRLFS-finder: a model, web server and stand-alone tool for prediction and analysis of R-loop forming sequences — SUPPLEMENTARY DATA 

# QmRLFS-finder: a model, web server and stand-alone tool for prediction and analysis of R-loop forming sequences

## SUPPLEMENTARY DATA

**Files in this Data Supplement:**

- SUPPLEMENTARY DATA
