# Supplementary material for: QmRLFS-finder: a model, web server and stand-alone tool for prediction and analysis of R-loop forming sequences
Source: Nucleic Acids Res. 2015 Apr 16;43(Web Server issue):W527–34. doi: 10.1093/nar/gkv344 (PMC4489302; doi:10.1093/nar/gkv344)
Supplement: SUPPLEMENTARY DATA [file supp_gkv344_nar-03708-web-b-2014-File005.docx]

**SUPPLEMENTARY DOCUMENT**

**DNA/RNA Immunoprecipitation assay (DRIP-qPCR)**

Genomic DNA and RNA were extracted from ovarian cancer cell line SKOV3 cells (HTB-77; ATCC, Manassas, VA). The cells were grown in RPMI with 10% FBS and maintained in 5% CO2 at 37°C. DNA and RNA samples were performed using the Allprep DNA/RNA isolation kit (Qiagen, USA) following the manufacturers' instructions. Genomic DNA extracts were digested overnight at 37oC with 50 U of HindIII, EcoRI, BsrGI, XbaI and SspI, with BSA. 40 µl of Protein G beads (50:50 slurry) (Millipore, Germany) was added to the extracts and pre-cleared to minimize non-specific binding. In a separate tube, 15 µg of anti-R-loop antibody (S9.6) and 40 µl of Protein G beads (50:50 slurry) (Millipore, Germany) were added together and incubated at 4°C overnight on a rotating wheel to allow binding of antibody to beads. The extracts were divided equally one was left untreated; other was treated with 10 U of RNase H (New England BioLabs, USA) as negative control. Extracts were incubated at 37°C for 2 h, heat inactivated for 20 min at 65°C then added on to the antibody-bound Protein G beads and incubated at 4°C overnight on a rotating wheel to allow binding of R-loops fragments to antibody-bound beads. The beads were pelleted at 2000 rpm for 5 min and the supernatant was discarded. The beads were washed once with the IP Wash Buffer 1 (20 mM Tris-HCl pH 8.1, 2 mM EDTA, 50 mM NaCl, 1% Triton X-100, 0.1% SDS), twice with the High Salt Wash Buffer (20 mM Tris-HCl pH 8.1, 2 mM EDTA, 500 mM NaCl, 1% Triton X-100, 0.1% SDS), once with the IP Wash Buffer 2 (10 mM Tris-HCl pH 8.1, 1 mM EDTA, 0.25 M LiCl, 1% NP-40, 1% Deoxycholic Acid) and twice with TE Buffer (20 mM Tris-HCl pH 8.0, 1 mM EDTA). The immunoprecipitates were incubated at 4°C on a rocker for 3 min between each wash. The nucleic acids were then eluted twice with 100 µl of Elution Buffer (100 mM NaHCO3, 1% SDS). 3 µl of Proteinase K (20 mg/ml) was then added to each sample and incubated at 55°C. DNA purified from excess salts and proteins and further estimated concentration by nanodrop.

**PCR analyses from DRIP assay**

Primers for specified gene set were designed using primer3 software and enlisted. qPCR experiments were conducted using QuantStudio™ 6 Flex Real-Time PCR system at initial denaturation at 95°C for 3 min, and further run at 40 cycles, denaturation step at 95°C for 15 sec, and extension step at 60°C for 40 sec. The values were initially normalized with loading input and estimated relative fold change abundance of DNA–RNA hybrid immunoprecipitated between untreated and RNase H treated sample at specified region. Primers used in DRIP-qPCR gene expression studies are listed in Supplementary Table 1.

**Supplementary Table 1.** Primers used in DRIP-qPCR gene expression studies

| **Gene** | **Forward primer** | **Reverse primer** |
| --- | --- | --- |
| *PTEN* | GTGATGTGGCGGGACTCTTT | ACAGCGGCTCAACTCTCAAA |
| *PBX1* | GCAGGGCACCCATCAGG | GCGGAGCTGTTACCTAATCCA |
| *JTB* | AGCTTGACGGTGAATCCTCG | GGCGCGTGTTTGTTCATTCT |
| *PPM1D* | AGCATCATGCCTGCTCTGA | GGCACAACAGAATGTTAATCCA |
| *TP53* | CTCCGTGATGCCTACCAAGT | CCCAGCCTGATTCCATTCTA |


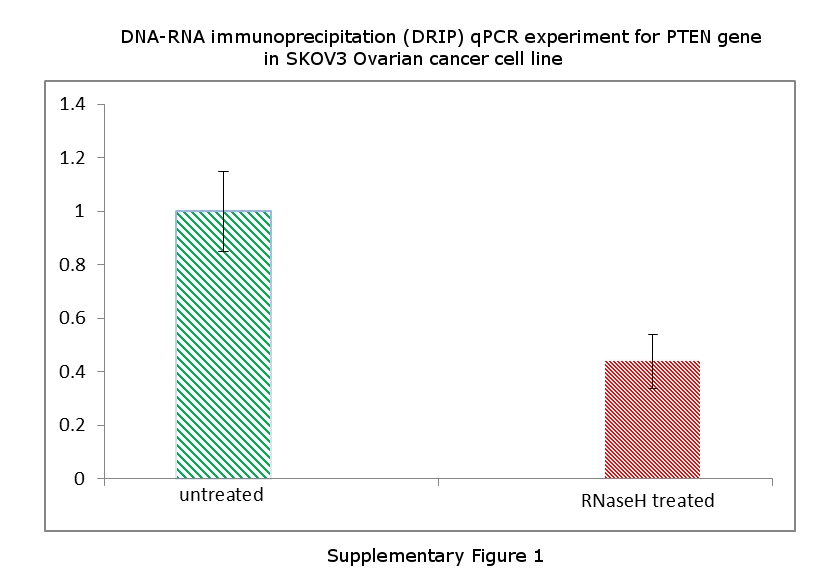


**Supplementary Figure 1.** Normalized expression values of the DRIP-qPCR experiment detected in untreated and RNase H -treated samples. This experiment provides a validation of computationally predicted RLFS-positive DNA segment located in the 5’UTR of PTEN gene expressed in the cells of ovarian cancer cell line SKOV3. The plot represents the results of DRIP-qPCR experiment validating the computationally predicted RLFS-positive DNA segment located in the 5’UTR of PTEN gene (Figure 2 C, Supplementary Table 2). Computationally predicted RLFS-negative loci selected in the TP53, JTB, and PPM1D genes were used as our negative controls (Supplementary Table 1). For all these loci the CT-value was greater than 32 (CT > 32) indicating lack of RNA-DNA hybrid formation in the both untreated and RNase H treated samples (results are not shown).

**Supplementary Table 2.** The delta CT value of *PTEN* gene

|  | CT | dCT | avg dCT | avg ddCT | Relative Fold | stdev |
| --- | --- | --- | --- | --- | --- | --- |
| *PTEN*_untreated | 27.209 | -1.2 | -1.34 | 0 | 1 | 0.1 |
| *PTEN*_untreated | 27.368 | -1.47 |  |  |  |  |
| *PTEN*_untreated | 27.399 | -1.35 |  |  |  |  |
| *PTEN*_RNaseH | 28.209 | -2.47 | -2.53 | 1.188 | 0.438 | 0.15 |
| *PTEN*_RNaseH | 27.937 | -2.51 |  |  |  |  |
| *PTEN*_RNaseH | 27.955 | -2.61 |  |  |  |  |

dCT is the value obtained after normalizing with loading control.

**Non-random mapping of the QmRLFS models and observed RLFS at a single gene level analysis**

In 21 of the 22 cases, the observed RLFS have been mapped into mammalian (human, mouse) genes. Currently, this is a set of all experimentally defined RLFSs/R-loops, detected and mapped by different methods at a single gene level in the mammalians. Taken into account that a typical span of RLFS is much smaller (about 270 bp) than a typical mammalian gene span (median of gene sizes are about 16,360 bp for mouse and 20,460 bp for human), it is very unlikely that the 20 of 21 computationally predicted RLFS loci (Table 1) can be co-localized with 20 independently detected RLFS loci located randomly within any of 21 selected mouse/human genes.

If so, the null hypothesis regarding to 20 of the 21 RLFS random co-localizations should be rejected. Actually, a probability of a co-localization of QmRLFS -predicted locus and a random sequence (with typical RLFS length) within a gene region can be estimated, for instance, for the 20 of 21 random humans and/or mice genes. Assuming at least one-nucleotide intersection, a probability of common region occurrence for a QmRLFS-localized sequence and a random sequence of 270- nt span (typical span of RLFS) within a human gene region is estimated as the following p=2*270/20,460=0.026393. In our analysis for Table 1, the number of opportunities for such co-localization event is 21 and the number of times that the event was actually occurred is 20. According to binomial probability function, such co-occurrence event could be observed by a chance with the probability 9.99x10^-16^. Using the 25,844 Refseq genes for multivariate (Bonferroni) correction the probability equals 2.58x10^-12^. Similar results can be obtained for RLFS in mouse genes.

**Performance evaluation**

We used three measures, accuracy, sensitivity and specificity to evaluate performance. They are defined below:

$$\boldsymbol{Accuracy=}\frac{\boldsymbol{TP+TN}}{\boldsymbol{TP+TN+FP+FN}}$$

$$\boldsymbol{Sensitivity=}\frac{\boldsymbol{TP}}{\boldsymbol{TP+FN}}$$

$$\boldsymbol{Specificity=}\frac{\boldsymbol{TN}}{\boldsymbol{TN+FP}}$$

In the formulas above, TP is the abbreviation of true positives (DNA sequences predicted to be RLFS that are supported by at least one experimentally defined R-loop); TN is the abbreviation of true negatives (DNA sequences predicted to be non RLFS that are not supported by experimentally defined R-loop); FP is the abbreviation of false positives (DNA sequences predicted to be non RLFS that are supported by at least one experimentally defined R-loop); FN is the abbreviation of false negatives (DNA sequences predicted to be RLFS that are not supported by experimentally defined R-loop).
